# Study protocol for the Danish High Risk and Resilience Study VIA 19 – from age 15 to age 19: transition from adolescence to early adulthood

**DOI:** 10.3389/fpsyt.2026.1848366

**Published:** 2026-08-17

**Authors:** Anne A. E. Thorup, Nicoline Hemager, Aja Neergaard Greve, Merete Birk, Carsten Hjorthøj, Martin Dietz, Oskar Hougaard Jefsen, Mette Falkenberg Krantz, Maja Gregersen, Lotte Veddum, Anja Alrø, Sanciya Mano Perfalk, Charlotte Sand Nielsen, Sofie Baltser Christensen, Mette Enevoldsen, Alberte Ehlig Høtoft, Nikoline Birkelund, Martin Wilms, Anne Søndergaard, Nanna Ørslev Weye, Line Bjørklund, Christoffer Brusen Barsøe, Claus Thorn Ekstrøm, Poul Jennum, Ron Nudel, Anna Starnawska, Kit Melissa Larsen, Kathrine Skak Madsen, William F. C. Baare, Vasileios Ioakeimidis, Adam Kaminski, Júlia Díaz-i-Calvete, Mathias Houe Andersen, Leif Østergaard, Hartwig R. Siebner, Ole Mors, Merete Nordentoft

**Affiliations:** 1 Child and Adolescent Mental Health Center, Research Unit, Copenhagen University Hospital, Capital Region of Denmark, Copenhagen, Denmark; 2 Department of Clinical Medicine, Faculty of Health and Medical Sciences, University of Copenhagen, Copenhagen, Denmark; 3 Department of Psychology, Faculty of Social Sciences, University of Copenhagen, Copenhagen, Denmark; 4 iPSYCH -The Lundbeck Foundation Initiative for Integrative Psychiatric Research, Aarhus, Denmark; 5 Copenhagen Research Center for Mental Health (CORE), Copenhagen University Hospital, Capital Region of Denmark, Copenhagen, Denmark; 6 Psychosis Research Unit, Aarhus University Hospital Psychiatry, Aarhus, Denmark; 7 Department of Clinical Medicine, Faculty of Health and Medical Sciences, Aarhus University, Aarhus, Denmark; 8 Section of Epidemiology, Department of Public Health, University of Copenhagen, Copenhagen, Denmark; 9 Center for Functionally Integrative Neuroscience, Department of Clinical Medicine, Aarhus University, Aarhus, Denmark; 10 Department of Child and Adolescent Psychiatry, Aarhus University Hospital Psychiatry, Aarhus, Denmark; 11 Biostatistics, Department of Public Health, University of Copenhagen, Copenhagen, Denmark; 12 Danish Center for Sleep Medicine, Department of Clinical Neurophysiology, Rigshospitalet, and Technical University Hospital, Copenhagen, Denmark; 13 Copenhagen Research Center for Biological and Precision Psychiatry, Copenhagen University Hospital, Copenhagen, Denmark; 14 Department of Biomedicine, Faculty of Health Sciences, Aarhus University, Aarhus, Denmark; 15 Center for Genomics and Personalized Medicine, CGPM, and Center for Integrative Sequencing, iSEQ, Aarhus, Denmark; 16 Danish Research Centre for Magnetic Resonance, Department of Radiology and Nuclear Medicine, Copenhagen University Hospital - Amager and Hvidovre, Copenhagen, Denmark; 17 Department of Regional Health Research, University of Southern Denmark, Hvidovre, Denmark; 18 Department of Clinical Medicine, Department of Radiology, Aarhus University, Aarhus, Denmark; 19 Department of Neurology, Copenhagen University Hospital - Bispebjerg and Frederiksberg, Copenhagen, Denmark

**Keywords:** developmental psychopathology, familial high risk, longitudinal observation, mental disorders, risk factors

## Abstract

**Background:**

Longitudinal clinical cohorts following individuals, who are born to parents with severe mental illness, from childhood into adulthood, provide invaluable insight into development of psychopathology, illness course over time and risk and resilience factors. Further, initiatives to prevent mental illness and support families at risk can be inspired and informed. The Danish High Risk and Resilience Study - the VIA cohort was founded during 2013-2015, where 522 7-year-old individuals born to parents with either schizophrenia bipolar disorder or controls, were recruited together with their parents and thoroughly examined. This study protocol presents the VIA 19 study, which is the fourth wave of the study investigating mental health and neurodevelopment in the 19-year-old participants, now in early adulthood.

**Method and design:**

Participants are invited to the clinic for a two-day assessment going through a clinical test battery, resembling that of the former waves and following up on previously selected major primary outcomes including daily functioning and social status, neurocognition, social cognition, mental health status, motor function, risk behavior among many other factors. Additionally, the young adults are invited to participate in neuroimaging, sleep assessment and provide samples of biological and genetic markers.

**Results:**

The data collection started in August 2024. A large effort is put into reaching all the young adults to ensure a high retention rate resembling the rates from the former waves ranging between 82 and 89%.

**Discussion:**

At age 19 we expect several full manifestations of first episode schizophrenia or bipolar disorder. Due to the longitudinal design, we will be able to draw trajectories of various kinds of outcomes and thus pinpoint developmental pathways from childhood to early adulthood.

## 1 Introduction

The saying ‘It runs in the family’ refers to the observation that mental illness may be caused by inherited gene variants, combined with the influence of growing up in a family environment affected by parental mental health problems. Since the early 1950s, familial high-risk (FHR) studies have shown that offspring of parents with mental health problems have increased risk of developing mental illness (1–3). These offspring have an increased risk of neurointegrative problems, social difficulties, poorer cognitive and motor skills, as well as early detectable psychiatric deficits¨ (1, 2, 4–7). A recent meta-analysis by leading experts in the field confirmed that this risk applies not only to children of parents with severe mental illnesses such as schizophrenia, bipolar disorder and depression but also to those with any psychiatric diagnosis (8). Furthermore, these studies have shown that offspring have an overall increased transdiagnostic risk beyond the diagnosis of their parent.

To provide a stronger basis for understanding disease mechanisms in transgenerational transmission and in general psychopathology from childhood and into adulthood, we established The Danish High Risk and Resilience Study – VIA (the VIA Study) in 2013. The VIA study is a population based, nationwide cohort study comprising 522 children born to parents with schizophrenia, bipolar disorder, or neither of these disorders (9, 10). This cohort is the first prospective study to provide comprehensive, multi-informant-based phenotyping of same-aged children every four years. The results from the first assessment at age seven, the VIA 7study, have already provided insight into new possible markers of early neurodevelopmental disturbances in children of parents with schizophrenia or bipolar disorder. The findings included deficits in neurocognitive functions (11), (subgroup only) (12), motor and social functioning (13), social cognition and language (14), olfactory function and visual speed (15, 16) as well as increased risk of mental disorders and psychotic-like experiences (17, 18) and poorer quality of life (19). Experiences of helplessness and social relationships were also described more negatively by caregivers in families with parental mental illness (20, 21). The findings from baseline at age 7 were largely confirmed at the second assessment at age 11 in the VIA 11 Study, indicating that the early-detected impairments were not merely developmental delays but rather stable indicators of early risk, at least from age seven to age eleven. We found that these children continued to more often live in inadequate home environments (22, 23) and with poorer family functioning (21), have more psychiatric symptoms (18, 24), poorer social functioning (14, 25), more cognitive impairments (26–28), and poorer motor skills (29). These results provided inspiration and knowledge to develop and initiate a study about innovative, preventive interventions (30). Preliminary, unpublished evidence from the third assessment wave, the VIA 15 study, indicate that the general patterns are not fundamentally changed regarding group differences in e.g. neurocognition, motor and psychopathology at age 15.

*Adolescence - The VIA 19 study*

Adolescence is generally defined as the period of transition from childhood to adulthood, unfolding from age 10 to age 19, with puberty encompassing a 2-5-year period within adolescence characterized by ongoing brain development, alongside marked changes in physical and bodily composition, hormonal status and psychological and social functioning (31, 32). Puberty ends around age 16-17, with boys a bit later than girls (33). While the VIA 7, 11 and 15 studies covered middle childhood at age seven and early and late puberty at ages 11 and 15, the VIA 19 study marks the end of adolescence and the beginning of young adulthood. This is an important period of life in terms of the continuous refinement of brain networks underlying especially executive function and emotional regulation. It is also characterized by increased psychological stability, independence from caregivers, identity consolidation, legal status, rights and obligations, formation of long term social, romantic and occupational roles, but also a period of impulsivity and risk taking behavior and increased prevalence of mental health disorders (34).

*Brain development* during adolescence is characterized by heightened plasticity, which enables the central nervous system to mature through large−scale reorganization and refinement of neural circuits. This process involves both synaptic pruning and increased axonal myelination, increasing the efficiency and speed of remaining neural pathways. Genetic factors, environmental influences, and their interactions play a critical role in shaping these developmental processes (35). A review from 2019 identified four core principles underlying typical adolescent brain development: 1) Neurodevelopment follows a non−linear trajectory; 2) brain networks become increasingly integrated and specialized, 3) an evolving imbalance between prefrontal executive control systems and limbic reward and arousal systems substantially influences behavior, contributing to heightened risk−taking as well as enhanced learning capacity that started even earlier in adolescence (the VIA 15 study) (36), and 4) this developmental plasticity entails increased vulnerability, reflected in heightened sensitivity to stress and elevated risk for the emergence of mental disorders (35).

Structural and functional brain abnormalities have been observed in drug-naïve adult patients with schizophrenia, and some of the strongest risk factors exert their influence already in the pre- or perinatal period (37). To our knowledge the VIA study is the first prospective cohort to acquire structural and functional magnetic Resonance Imaging (MRI) data from a large age-controlled cohort of children at familial high-risk assessed from childhood to puberty. This design allows repeated within-subject mapping of neurodevelopment across this sensitive period of brain maturation. Findings from the neuroimaging baseline in the VIA 11 cohort have shown both structural and functional brain differences in children at familial high risk. Specifically, we have observed sex-specific differences in cortical brain volumes (38), and aberrant functional connectivity within the mentalizing network during a social cognition task (39). In contrast, no group differences were found in task- related brain activation during cognitive control as assessed with the Flanker task (test of selective attention and inhibitory control) (40).

Both schizophrenia and bipolar disorder are associated with abnormalities in sensory and perceptual responses of the cerebral cortex, as measured with magnetoencephalography (MEG) and electroencephalography (EEG). Several studies of clinical high-risk individuals and family members of affected individuals demonstrate similar deficits, indicating that EEG and MEG markers may both be used as predictive biomarkers and provide insight into the pathophysiology and development of mental disorders (41–44). Using biophysical models of the cortical networks, dynamic causal modelling (DCM) can be applied to infer deficient synaptic mechanisms within the cortical circuits, bridging the translational gap between non-invasive human neuroimaging and invasive animal models of brain disorders. Data from the first imaging wave at age 11 showed comparative responses, within these domains, however using models of connectivity we showed altered patterns echoing findings from individuals with manifest schizophrenia (45). In a recent study of offspring with familial risk for schizophrenia and bipolar disorder, the analysis of a spatio-temporal connectome has revealed prominent group differences in brain organization, comparing the same vulnerable groups as our study, however within a broad age range, and a considerably smaller sample (46).

Aberrant modulations of neural connectivity within central areas of the brain, may increase the individual’s vulnerability to a neurodevelopmental disease. By merging two types of neuroimaging data, e.g. diffusion-weighted imaging (DWI), functional MRI (fMRI), EEG and MEG, we will be able to perform multimodal analysis of brain connectivity and their trajectories. This enables examination of how networks segregate into well-known functional systems and to map the preferred routes and patterns of activity propagation.

*Mental health* problems are common in this period of life, incidence rates for several psychiatric diagnoses rise, peaking in late adolescence and early adulthood (47). Given that this developmental period encompasses the emergence of independence from parents, educational attainment, and identity formation, this period is crucial for the understanding of youth mental health development and psychopathology. *Neurodevelopmental disorders* such as attention-deficit/hyperactivity disorder (ADHD) and autism spectrum disorders are often diagnosed in childhood or early adolescence and psychoeducation and insight combined with various kinds of treatment may influence symptom severity and function positively. However, the adolescent brain undergoes maturational changes that may also impact the expression of for example impulsivity and hyperactivity (35). The frequent *use of psychoactive substances* in adolescence, especially alcohol, cannabis and nicotine (48), are considered a well-established independent risk factor for later development of neuropsychiatric disorders, since drugs can affect the developmental processes, structure and networks in the brain and functions like behavior and cognition negatively. Of special concern is the research indicating a possible relation between particularly intake of cannabis in early adolescence and later development first episode psychosis and schizophrenia (48). For many adolescents the ability to handle emotional changes improves considerably during adolescence, while for some it becomes increasingly difficult and even marks the onset of psychopathology (e.g., depression). From early in life, caregivers have played a crucial role for the child in recognizing and managing emotions, and they thus lay the ground for the child’s ability to learn to self-regulate. Later, young people rather turn to their peers for emotional processing and regulation (49). Therefore *social relations* with peers become increasingly important during adolescence, while social interest in parents and younger children decreases (32). The adolescent brain development is not linear, and some systems develop more rapidly than others, which may be part of the explanation as to why adolescents are willing to take risks when being with same-aged peers (50). *Becoming sexually active* is also an important area of development in adolescence, since it is often the time for first romantic relationship which may both be an exciting challenge, a stress factor, a positive event and provide important life experiences. Some become pregnant, and some are parents themselves. Further, an increasing number of adolescents in society seek help for gender dysphoria (51). At age 19 the participants are of legal age and may have left their parents’ home and live on their own or they may still be living with one or both parents. Developmental milestones of ability to take care of oneself, manage financial matters and have a meaningful daily activity while also handling social relations are important measures to understand young adulthood outcomes. *Technology and social media* have been implemented widely in society during the decade, when our cohort grew up, and this has led to theories and research about the potential impact on youth mental health, and their thriving, social connectedness, and physical health as well. The field is extremely complex, and most research indicates mixed results regarding associations between digital use and well-being among adolescents (52).

## 2 Aims and hypotheses

The overarching aim of the fourth wave of The Danish High Risk and Resilience Study - VIA 19 is to follow up on the earlier-defined domains of development and function in the now 19-year-old participants to elucidate and characterize developmental trajectories that determine mental health. The domains of interest are psychopathology, neurocognition, motor function, social cognition and functioning, structural and functional brain maturation (MRI, EEG, MEG), and environmental risk assessment, including family circumstances and risk behavior.

### 2.1 Specified aims

1. To improve insight into early disease processes in schizophrenia and bipolar disorder, including early symptom formation, prodromal states and signs of psychopathology, motor functioning, neurocognitive, social cognitive and social functioning impairments, and maturational delays in the domains of cognitive functioning, including social cognition, and how these are related to structural and functional changes in the brain.
2. To identify the influence of genetic, epigenetic, and environmental exposures by analyzing associations between outcomes such as psychopathology, risk behavior, and social and cognitive functioning and exposures such as polygenic scores (PGSs). Environmental factors include, among others, direct measures of the emotional climate in the family. In terms of PGSs, we have computed scores for disorders ascertained in the parents, namely, schizophrenia and bipolar disorder, based on the largest currently available genome-wide association studies. Additionally, we have computed scores for related disorders such as major depressive disorder and autism spectrum disorder, both of which may overlap with the ascertained disorders in clinical presentation and in genetic etiology or may be co-morbid with them (53–56), as well as for related traits such as educational attainment, which is known to be lower in schizophrenia (57). Using these PGSs, we will be able to control for genetic predisposition to other disorders (as appropriate in a given analysis) as well as study the genetic relationship between different genetically-influenced traits and the traits measured in our study.

## 3 Method and design

The majority of methods has been described in detail in the VIA 15 study protocol (36). For the reader’s convenience, these descriptions are reproduced here and properly referenced.

### 3.1 Design

**Figure 1:**
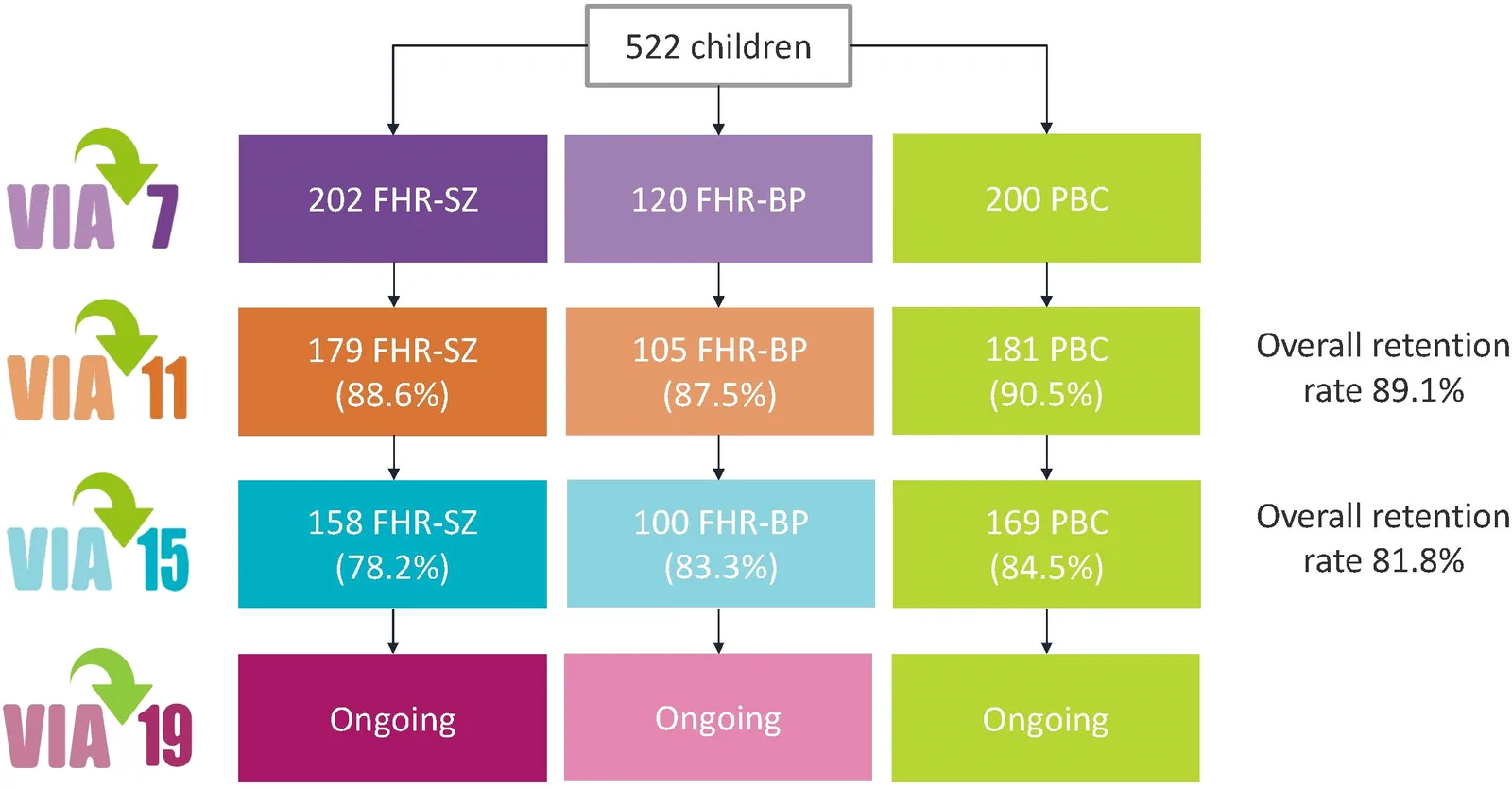
Flowchart of retention rates over time.

The Danish High Risk and Resilience Study is a population-based, longitudinal multi-informant cohort study consisting of 522 children born to parents with schizophrenia, bipolar disorder or neither of these disorders. The participating children and their parents were recruited from Danish population-based registers and investigated thoroughly during 2013-2015, when the children were seven years old. The first assessment is referred to as the VIA 7 study (9). The second wave of assessments, the VIA 11 study, was carried out when the children were 11 years of age from 2017-2020, completed with a participation rate of 89.1 percent (58). The third wave of assessment, the VIA 15 study (36), was carried out when the children were 15 years of age from 2021 to 2024 with a retention rate of 81.8%. (see **Figure 1**, flowchart).

The cohort at baseline consisted of:

- 202 children with at least one parent diagnosed with schizophrenia spectrum psychosis
- 120 children with at least one parent diagnosed with bipolar disorder
- 200 children with neither of the parents treated in mental health services for the above diagnoses.

The population-based control children (PBC) were matched to children at familial high-risk of schizophrenia (FHR-SZ) on municipality, assigned sex at birth, and age (+/- 3months). We included children at familial high-risk of bipolar disorder (FHR-BP) as a non-matched group; however, the group was comparable to the two other groups with respect to age and sex (10). The representative cohort is based on data from The Danish Civil Registration System (59) linked to the Danish Psychiatric Central Research Register (60).

*Earlier assessments*: At all former waves of data collection, the children and their parents were thoroughly examined with diagnostic interviews, neuro- and social cognitive tests, questionnaires, home visits and observations. MRI scans were performed at the VIA 11 study and the VIA 15 study. Assessments were supplemented with data from questionnaires sent to schoolteachers in all three waves. Outcomes for the children were assessed thoroughly in the domains of neuromotor functioning, neuro- and social cognitive functioning, social functioning, and psychopathology at all ages. Also, parents were interviewed about their own mental health and data on their neurocognitive functioning were collected at baseline, the VIA 7 study. In the VIA 11 study parents were interviewed about their mental health and about their personal daily functioning during the previous month. Sociodemographic data was collected in both VIA 11 and VIA 15 studies by caregivers, while in VIA 15 the adult caregiver was only interviewed about function within the previous month. All assessors were kept blinded to whether the children were at familial high-risk or were population-based controls. The full assessment batteries in the VIA 7, the VIA 11 and the VIA 15 study lasted approximately three days, and most of the families completed the whole battery (see flowchart). Parents were always offered verbal feedback on their child’s performance and in the VIA 15 study, this was also offered directly to the adolescent. All participants received a gift card for their time taken and practical obstacles like transportation and catering were organized by the researchers and expenses were covered by the VIA study. All individuals remain in the cohort and in the group, they were originally assigned to, regardless of if they are diagnosed with a mental disorder at any time.

A small proportion of the families who participated in the VIA 7 study expressed that they did not wish to participate in the VIA 11 and/or VIA 15 study for various reasons. Some parents indicated that they would never like to participate again and were excluded from all future invitations, while others expressed that we willing to be contacted again for future assessments and that they only declined participation in the specific wave. This information is all carefully registered in our cohort files. For the VIA 19 study, however, the participants are young adults, who are now of legal age and able to make their own decisions. Therefore, we will invite all 522 participating offspring from the baseline cohort to participate in the VIA 19 study. Data from the VIA 7 study is stored at Statistics Denmark and linked to register-based information about use of mental and somatic health services for parents and children (National Patient Register (60, 61), parental education and source and level of income (62). Data from the VIA 11 study, the VIA 15 study, and the VIA 19 study will also be stored at Statistics Denmark.

### 3.2 Method of assessment

The test battery in the VIA 19 study is solely directed toward the young adult, and parents will not be assessed. However, a ‘former caregiver’ will be identified, this may be a biological parent or another adult, who either lives or lived with the young adult and has been responsible for the young adult’s wellbeing on a regular basis during the previous four years and who knows the young adult best at present. This former caregiver will be asked to fill in questionnaires about the young adult’s social functioning, social responsiveness and executive functions. If the young adult does not wish to participate in the VIA 19 study but gives written consent for the former caregiver to be contacted, the latter will also be asked to fill out an anamnestic questionnaire about the young adult’s life course the preceding four years since, (i.e., time from the VIA 15 study and up till the VIA 19 study).

The VIA 19 test battery takes approximately three days to complete, two days in the research clinic and one day with brain scan (5–6 hours duration per day including breaks), and a home visit for those who accept to also participate in the sleep assessment with polysomnography (PSG) for one night. An anamnestic interview will cover received treatment for mental health problems and support for living situation, school, education, job etc. from health and social service systems. Flexibility in terms of place and time is key. Some may find it exhausting, but individual needs (e.g., for breaks or short test days) will always be considered to ensure a positive experience for the participating young adult. All outcome measures are examined with validated instruments that are specifically developed and selected for this age group. Many variables will be measured for the fourth time, enabling trajectory analyses. The battery is comprehensive and consists of both interviews, tests, observation, and questionnaires (see section below and table for details). All researchers will be blinded to the familial risk status of each participant, but will have access to data from former waves regarding the young adult’s mental health, cognitive functioning, etc.

### 3.3 Overview of domains and instruments in the VIA 19 study

#### 3.3.1 Neuromotor function, biological measures, and physical health

Manual dexterity, ball skills and balance are assessed with the Movement Assessment Battery for Children-2 (Movement ABC-2) (63), which is a clinical test for motor function also for adolescents (64). The impact of gross and fine motor difficulties on daily life, and non-motor factors affecting movement is examined using the self-report “Movement Assessment Battery for Children, Third Edition (Movement ABC-3) Checklist” (65). The self-report is complementary to the clinical test. For those young adults who will participate in brain mapping at and the Danish Research Centre for Magnetic Resonance (DRCMR), distal fine motor movements of right and left hand will be assessed with a circle drawing task using a drawing tablet and CSWin software. Fine motor coordination, hand dexterity and finger movement speed will be assessed with the Nine-Hole Peg Test [9-HPT (66)].

We will make a clinical evaluation of anthropometry of the young adult (height, weight, and waist circumference) in the clinic. Further, a dried blood spot on filter paper and a saliva sample will be collected for later analysis of genetic and epigenetic analyses. Physical activity will as in the VIA 11 and the VIA 15 study be measured by an activity sensor in an easily wearable adhesive patch (SENS motion^®^) (67), which directly measures level of physical activity during a one-week observation period. Bodily distress symptoms are covered by the questionnaire Body Distress symptoms checklist (BDS) (68) and screening for somatization and hypochondriasis is covered by the questionnaire Whiteley Index 6-R (WI-6) (69).

Smell identification is measured with the Brief Smell Identification Test (B-SIT) (70).

#### 3.3.2 Neurocognitive function and language

The neurocognitive test battery includes Rey’s Complex Figure Test (71), Rapid Visual Information Processing (from the Cambridge Neuropsychological Test Automated Battery (CANTAB) (72), Verbal Fluency Phonemic, Semantic and Switching and Trail Making Test Number Sequencing, Letter Sequencing and Number-Letter Switching from the Delis-Kaplan Executive Function System (D-KEFS) (73), Symbol Search and Coding from the Wechsler Adult Intelligence Scale – Fourth Edition (WAIS-IV) (74) Stockings of Cambridge, Intra-Extra Dimensional Set Shift, and Spatial Working Memory (CANTAB) (72), Letter-Number Sequencing and Arithmetic (WAIS-IV) (74) and Reynolds Intellectual Screening Test (RIST) (75). Expressive language is assessed with the Recalling Sentences test (CELF-5) (76)^1^.

#### 3.3.3 Social cognition

Social cognition is measured with Animated Triangles (77) Emotion Recognition Task from the CANTAB (72), and the Social Cognition paradigm from the Human Connectome Project (78).

#### 3.3.4 Psychopathology

General psychopathology will be examined with a semi-structured, diagnostic interview: the Schedules for Clinical Assessment in Neuropsychiatry (SCAN) version 2.1 (79, 80). In connection with the SCAN interview, and as in the former waves, participants are interviewed thoroughly about any subthreshold psychotic experiences, (PE) including information about frequency, intensity, degree of conviction, and functional impact of these transient or irregular hallucinational experiences or delusional thoughts. Each symptom is given a final score on a seven-point scale, assimilating the Scale of Prodromal Scale (the SOPS scale), ranging from 0 to 6 (0=absent, 1=possibly present, 2=mild, 3=moderate, 4=moderately severe, 5=severe but not psychotic, 6=severe and psychotic). All diagnostic information including data on PE is presented at weekly clinical conferences chaired by a general adult psychiatrist and/or a child and adolescent psychiatrist (OM, MN, AT) concluding consensus diagnoses in DSM-IV and DSM-5 and International Statistical Classification of Diseases and Related Health Problems 10th Revision ICD-10 DCR (Diagnostic Criteria for Research). The SCAN 2.1 interview does not cover neurodevelopmental symptoms and behaviors, i.e. ADHD and autism spectrum disorders, therefore Section 1 in the forthcoming SCAN 3.0 (81) was added to the SCAN 2.1-interview. The SCAN interview does not cover personality disorders. (80).

Connors’ Adult ADHD Rating Scales (CAARS) (82) – Informant version is filled out by the close relative and the Adult Self Report Scale, ASRS (83) by the young adult to assess symptoms of ADHD. Affective lability will be measured using the Affective Lability Scale (ALS) (84). We also include Youth Experience Tracker Instrument (YETI) (85), a brief self-report measure designed to facilitate early identification of risk for severe forms of mental illness, including major depressive disorder, bipolar disorder, and schizophrenia.

By using the White Noise Paradigm (86), we will be able to investigate some of the young adults are more likely than others to appraise an ambiguous situation as threatening. We will apply a Danish version of the White Noise paradigm, which is a series of 75 very short sound clips in which in 50 of them short and neutral sentences are included in the sound of the white noise The respondents can select between five responses:’hearing positive/negative/neutral/voice/no speech/uncertain.

Deliberate self-harm is an interview developed by our own research group in collaboration with specialists in the area by collapsing items from two longer questionnaires (parts of Deliberate Self-Harm Inventory – Youth Version (87), and the Clinician-Administered Non-Suicidal Self-Injury Disorder Index (CANDI) (88). The interview was also used in VIA 15 and is adapted to the VIA 19 study by incorporating substantial revisions to questions concerning digital self-harm, which is a relatively new phenomenon and, therefore a rapidly evolving field of research (89). Bullying is assessed with a brief semi-structured interview based on a questionnaire by Olweus (90).

Executive functioning, including emotion regulation and flexibility, will be assessed with the Behavior Rating Inventory of Executive Function questionnaire (BRIEF) (91) by the former caregiver. We will also include BRIEF-Self-Report form which will allow us to assess self-reported executive functioning. Autism spectrum traits are evaluated with the Social Responsiveness Scale, Second Edition (SRS-2) (92) completed by both the young adult and the former caregiver. Adverse life events including unwanted sexual experiences will be assessed by the questionnaire, Childhood Trauma Questionnaire (CTQ) (93, 94).

#### 3.3.5 Social functioning, self-esteem, risk-taking behavior, loneliness, social isolation, and resilience

The current social functioning of the young adult will be assessed using the Personal and Social Performance Scale (PSP), a semi structured interview containing four domains (95).

Development of social functioning is captured by the Vineland-2 rating form for parents/caregivers (96).

Risk-taking behavior will be assessed with a modified and adapted descriptive questionnaire based on Youth Risk Behavior Surveillance System (97), as applied in VIA 15. Due to outdated formulations, particularly in questions and language about gender identity and sexuality, and the fact that the participants are now adults, some of the questions have been rephrased or revised to be more age appropriate. In addition, some questions are new (e.g. regarding pregnancy, abortion and venereal diseases). Inspiration is derived from the Danish LGBT+ website and reports from a recent Danish research project called ‘Projekt SEXUS’ regarding sexuality in the population and reports from The National Board of Health, which is the highest healthcare authority in Denmark (98, 99).

An anamnestic interview is used to gather information on housing conditions, primary occupation, potential parenthood, social relationships, leisure activities, and stressors experienced since the VIA 15 study, and include a brief assessment of physical and mental health as well as any treatment received during this period. Perceived social support will be assessed with the questionnaire, Multidimensional Scale of Perceived Social Support (MSPSS) (100), and self-esteem is covered with Rosenberg Self-Esteem Scale (RSE) (101, 102).

The Three-Item Loneliness Scale (T-ILS), which is a short version of the Revised University of California Los Angeles Loneliness Scale 20 items (R-UCLA Loneliness Scale) (103), assesses loneliness and social isolation (104) and is filled in by the young adult. Social isolation will also be measured with an index based on 6 questions regarding social contact inspired by the more comprehensive Social Network Index (105) and adapted by senior researcher M. Lasgaard. Leisure time screen-based media use will be assessed using six items from the SCREENS questionnaire (106), while resilience is measured by Child and Youth Resilience Measure - revised (CYRM-R) (107) (translation by Kis & Koldsø, 2021).

#### 3.3.6 Multimodal brain imaging

We will repeat the brain imaging procedures performed at ages 11 and 15, including anatomical and functional whole-brain MRI at 3.0 Tesla, EEG, and MEG (not carried out at age 11). MRI with harmonized acquisition parameters will be performed at the Center for Functionally Integrative Neuroscience (CFIN), Aarhus University Hospital and the Danish Research Centre for Magnetic Resonance (DRCMR), Hvidovre Hospital, Denmark. We will acquire T1-weighted images using a Magnetization Prepared 2 Rapid Acquisition Gradient Echoes (MP2RAGE) sequence, T2-weighted images and high-quality diffusion MRI (dMRI) data to derive brain morphological measures, myelin-sensitive maps (from the R1 maps from the MP2RAGE sequence) and microstructural properties, such as fractional anisotropy and apparent diffusion coefficient. Using well-established experimental paradigms, we will acquire functional brain activity and connectivity data while participants perform the Eriksen Flanker task (108), a reward paradigm and the social cognition paradigm from the Human Connectome Project (39, 78, 109, 110). Applied jointly, the three tasks probe executive cognitive control (i.e., ‘distractor resistance’ during fast response choices cued by directional visual cues), social cognition (i.e., inferring the social interactions of moving objects), and the reward system. We have chosen these tasks because the brain networks that mediate these tasks are hypothesized to be implicated in the pathophysiology of neurodevelopmental disorders. Functional profiling of these brain systems will enable us to infer specific network properties and dynamics that contribute to disease formation or resilience.

We will also repeat two auditory stimulus paradigms while participants undergo EEG (only at DRCMR, Copenhagen) and MEG (only at CFIN, Aarhus): an auditory oddball paradigm to measure the mismatch negativity (MMN) (111), as well as the auditory steady-state response (ASSR) (41). In addition, we record resting-state EEG and MEG. Like the fMRI part, we will repeat the Eriksen Flanker paradigm in EEG to investigate how the brain monitors and performs motor action with a cognitive challenge. The longitudinal perspective will contribute to increased understanding of the relationships between more basic functions and higher cognitive functions throughout adolescence, and hence the trajectories of self-regulation across groups.

By combining fMRI and EEG data (although not acquired concurrently) we will be able to get a deeper understanding of lower order processing as well as the interaction of specific brain regions during the emergence of psychopathology on one hand and cognitive control on the other during this age period.

#### 3.3.7 Polysomnography

PSG is a non-invasive EEG-based method, that was also used in a large subsample in VIA 15 (N = 248, paper in review). It is considered the gold standard of sleep analysis, and widely applied both in clinical practice as well as for research purposes (112). Except for an occasionally mild discomfort from sleeping with the equipment, there are no known side effects or complications to the method. We will examine the sleep macro and microstructure of young adults with PSG. For PSG recordings, we will use Somnomedics SomnoHD devices (https://somnomedics.de/en/solutions/sleep_diagnostics/stationary_sleep_lab_psg/somno_hd/), with an attached 32EEG head box. The EEG-montage is a 21-electrode setup with electrodes positioned according to the AASM international guidelines of the 10–20 system (113, 114). In addition to electroencephalographic signals from the scalp, we will record electrocardiographic signal from the chest, electromyographic signals from the chin and electrooculographic signals from the outer lateral canthi. Trained personnel will fit the PSG-equipment in the young adult’s home or in the clinic. The young adult will wear the PSG equipment for the duration of one night in their home. Next morning, after the recording, the young adult will remove the equipment and store it for collection by our staff. Prior to the PSG recording, the young adult will complete the Pittsburgh Sleep Quality Index (115). A finger-tapping motor sequence task was included to measure improvement in overnight motor skill performance, administered on the evening of the polysomnography assessment and retested the following morning (116). The data will be controlled for data quality and analysis will include manual and automated annotations in accordance with international standards. An expert at Danish Center for Sleep disorders, University Hospital of Copenhagen, Rigshospitalet, Prof. P. Jennum will score the expression of the various sleep stages based on the complete recording period, to produce a hypnogram for each participant. The morphology and temporospatial dynamics of sleep micro events including spindles (117) and K-Complex’ (118) in the EEG recording will be marked for each participant as well using well validated automated tools.

#### 3.3.8 Genetic and epigenetic analyses

In the VIA 7 study, saliva samples from the children and blood samples from the parents were used for genotyping on the Illumina PsychChip, and further markers were subsequently imputed for individuals passing quality control, as described previously (119). The genetic data have been used both in discovery analyses in the VIA cohort and in analyses of polygenic scores.

In the VIA 19 study, we will assay DNA methylation for those of the 522 children who provided written consent to use samples of the dried bloodspots from the Danish Genetic blood spot screening program for genetic disorders collected at birth and samples from all four follow-up visits (saliva and/or blood in the VIA 7, VIA 11, VIA 15 and VIA 19 studies). DNA methylation is a well-established and stable epigenetic marker, providing insights into how environmental and biological factors influence gene regulation over time, and offering unique opportunities to identify molecular mechanisms underlying health and disease trajectories. This will allow us to provide a longitudinal assessment of epigenetic changes from birth through childhood and adolescence.

Genome-wide DNA methylation will be assayed with the use of Infinium Methylation EPIC BeadChip (tagging over 900,000 sites across the genome). The epigenetic data will be subjected to stringent quality control and data processing using well-established Bioconductor packages (120–122). In order to account for cellular heterogeneity and reduce the confounding in the sample we will predict cell proportions from the epigenetic data and further adjust for these measures in our association models (123).

All assessments are listed in **Tables 1**, **2**.

**Table 1:** Assessment battery for the young adults participating in The Danish High Risk and Resilience Study VIA 19, all domains.

Domains	Outcomes	Instrument	Type of test	Approximate duration	In VIA 7	In VIA 11	In VIA 15
Neuromotor, biological measures and physical health	Motor function: manual dexterity, aiming & catching and balance	Movement Assessment Battery for Children-2 (Movement ABC-2)	Test	45 min	Yes	Yes	Yes
	Impact of gross and fine motor difficulties on daily life, and non-motor factors affecting movement	Movement Assessment Battery for Children-3 (Movement ABC-3)	Questionnaire	10 min	No	No	No
	Anthropometry	Measure of height, weight, waist	Measurement in clinic	5 min	Yes	Yes	Yes
	Physical activity and sleep	SENS chip	Chip on thigh for one week	5 min + 1 week	No	Yes	Yes
	Sleep Quality	Pittsburgh Sleep Quality Index (PSQI)	Questionnaire before PSG	5-10 min	No	No	Yes
	Bodily distress	Body Distress Symptoms checklist (BDS)	Questionnaire	2 min	No	No	Yes
	Health anxiety and somatization	Whiteley Index 6-R (WI-6)	Questionnaire	1 min	No	No	Yes
	Smell identification	Brief Smell Identification Test (B-SIT)	Test	10 min	Yes	No	Yes
	Distal fine motor movements of right and left hand	Circle drawing task	Test before MRI	5	No	Yes	Yes
	Fine motor coordination, hand dexterity and finger movement speed	Nine-Hole Peg Test (p-HPT)	Test before MRI	3	No	Yes	Yes
Neurocognitive function and language	Verbal Memory and visual memory	Word Selective Reminding from the Test of Memory and Learning Second Edition TOMAL-2	Test	10 min	Yes	Yes	Yes
		Figure Test (RCFT)	Test	8-10 min	Yes	Yes	Yes
	Attention	Rapid Visual Information Processing (RVP) from CANTAB	Computer test	10-15 min	Yes	Yes	Yes
	Flexibility and processing speed	Trail Making Test 2-4 from Delis-Kaplan Executive Function System (D-KEFS)	Test	8 min	Yes	Yes	Yes
		Symbol Search and Coding test from Wechsler Adult Intelligence Scale, Fourth Edition (WAIS-5)	Test	5 min	Yes*	Yes*	Yes*
		Verbal Fluency 1-2 from Delis-Kaplan Executive Function System (D-KEFS)	Test	4 min	Yes	Yes	Yes
	Executive functions (planning and flexibility)	Stockings of Cambridge (SOC) and Intra-Extra Dimensional Set Shift (IED) from CANTAB	Computer test	20-30 min	Yes	Yes	Yes
		Verbal Fluency 3 from Delis-Kaplan Executive Function System (D-KEFS)	Test	2 min	Yes	Yes	Yes
	Executive functions (error monitoring)	Flanker Task	Test before and during fMRI		No	Yes	Yes
	Executive functions (visual and verbal working memory)	Spatial Working Memory (SWM) from CANTAB	Computer test	10-15 min	Yes	Yes	Yes
		Letter-number Sequencing and Arithmetic from Wechsler Adult Intelligence Scale, Fourth Edition (WAIS-5)	Test	10-15 min	Yes*	Yes*	Yes*
	Intelligence	Reynolds Intellectual Screening (RIST)	Test	15 min	Yes	Yes	Yes
	Expressed language	Recalling Sentences test (CELF-5)	Test	5 min	No	No	No
Social cognition	Social cognition	Human Connectome Project Social Cognition Paradigm	Test during MRI	10 min	No	Yes	Yes
	Social cognition	The Animated Triangles Task	Test	12 min	Yes	Yes	Yes
	Emotion recognition	Emotion Recognition Task (ERT) from CANTAB	Computer test	11 min	Yes*	No	No
Psychopathology	Psychiatric symptoms	Schedules for Clinical Assessment in Neuropsychiatry, SCAN, version 2.1	Interview	45-120 min	Yes*	Yes*	Yes*
	Psychotic experiences	PE Hallucinations and PE Delusions	Interview inspired from the SOPS scale	1-10 min	Yes	Yes	Yes
	Attention/hyperactivity	Adult Self Report Scale (ASRS)	Questionnaire	5 min	No	No	No
	Affective liability	Affective Liability Scale (ALS)	Questionnaire	5 min	No	No	No
	Risk factors for mental illness	Youth Experience Tracker Instrument (YETI)	Questionnaire	3-5 min	No	No	Yes*
	Speech illusions	White Noise Test	Test	20 min	No	No	Yes*
	Self-harm	Semi structured interview adapted to use in VIA 15 with items from Deliberate Self-Harm Inventory Youth Version (DLSH-I) and Clinician-Administered Non-Suicidal Self-Injury Disorder Index (CANDI)	Interview	5-10 min	No	No	Yes*
	Executive functions	Behavior Rating Inventory of Executive Function (BRIEF)	Questionnaire	10 min	No	No	No
	Autism spectrum traits	Social Responsiveness Scale (SRS-2)	Questionnaire	10 min	No	No	No
	Adverse life events	Childhood Trauma Questionnaire-Short Form (CTQ- SF)	Questionnaire	3-5 min	No	No	Yes
Social function and behavior	General functioning	the Personal and Social Performance Scale (PSP)	Interview	20 min	Yes*	Yes*	Yes*
	Risk-taking behavior	Adapted questionnaire from 2019 National Risk Behaviour Survey	Questionnaire	5-10 min	No	No	Yes*
	Education, living situation, stressors, health and social life	Anamnesis	Interview or questionnaire	30-40 min	No	No	No
	Perceived social support	Multidimensional Scale of Perceived Social Support (MSPSS)	Questionnaire	2 min	No	No	Yes
	Self-esteem	Rosenberg Self- Esteem Scale (RSE)	Questionnaire	2 min	No	No	No
	Loneliness	The Three-Item Loneliness Scale (T- ILS)	Questionnaire	1 min	No	No	No
	Social network and contact	Social contact questionnaires	Questionnaire	2 min	No	No	Yes
	Bullying	Semi structured interview based on Olweus Bully/Victim Questionnaire	Interview	1-10 min	No	Yes	Yes
	Resilience	Child and Youth Resilience Measure Revised (CYRM-R)	Questionnaire	1-3 min	No	Yes*	Yes*
	Screen-based media time	SCREENS	Questionnaire	2 min	No	No	No
Brain mapping	Brain structure and brain activity	Structural and functional MRI. T1-, T2, and diffusion-weighted MRI. fMRI paradigms: Eriksen Flanker task, reward task, and social cognition task and EEG/MEG	Brain scan at hospital	90 min	No	Yes	Yes
	Electroencephalography, EEG (only at Copenhagen site)	40 Hz auditory steady state response Mismatch negativity Modified Eriksen Flanker task Resting state	Brain scan at hospital	120 min	No	Yes	Yes
	Magnetoencephalography, MEG (only at Aarhus site)	Paradigms: Roving auditory oddball + 40 Hz auditory steady state response Resting state	Brain scan at hospital	90 min	No	No	Yes
	Polysomnography, PSG	Polysomnogram	PSG monitor	Overnight	No	No	Yes
Genetic and epigenetic analyses	Epigenetics	Saliva sample, dry blood spot	Blood and saliva sample	5-10 min	Yes	Yes	Yes
	Genome-wide DNA methylation	Saliva sample, dry blood spot	Blood and saliva sample	5-10 min	No	Yes	Yes

* = The instrument has been changed or adapted to suit the age of the participants in VIA 19.

**Table 2:** Assessment battery for the former caregiver in The Danish high risk and resilience study - VIA 19.

Domains	Instrument	Type of test	Duration	In VIA 7	In VIA 11	In VIA 15
Education, living situation, stressors, health and social life	Anamnesis	Questionnaire	10 min	Yes**	Yes**	Yes**
Attention/hyperactivity	Rating Scales (CAARS) CAARS Informant	Questionnaire	5 min	Yes*	Yes*	Yes*
Executive functions	Behavior Rating Inventory of Executive Function (BRIEF)	Questionnaire	10 min	Yes*	Yes*	Yes*
Autism spectrum traits	Social Responsiveness Scale (SRS-2)	Questionnaire	10 min	No	Yes	Yes
Social development	Vineland Adaptive Behavior Scales II	Questionnaire	10 min	Yes	Yes**	Yes**

*The instrument has been changed or adapted to suit the age of the participants in VIA 19.

**The instrument has changed from an interview to a questionnaire.

### 3.4 Practical issues and ethical considerations

As in previous waves, we make a great effort to meet each participant with a friendly and flexible approach when arranging their participation. Thus, the assessment can last more than the standard three days and take place at times and places (except for MRI, EEG and MEG) that accommodate the young adult’s needs and preferences. If there are any specific tests, interviews, or questionnaires that the young adult does not wish to take part in, it will not lead to exclusion from the rest of the study. Transportation and catering are arranged in collaboration with each participant, and costs are covered. The young adult will receive a gift card of 1000 DKK for each completed nominal test day, a gift card for 500 DKK for participating in polysomnography, and up to 1200 DKK for participating in brain mapping (1000 DKK, with the possibility of earning an additional 200 DKK in the reward paradigm). The participants can receive a maximum of 3700 DKK in total, also if testing is spread over more than three days. The remuneration is taxable.

The young adult will be offered verbal feedback on the main conclusions when assessments are completed. Participation in the study does not include any interventions or treatment. In cases of obvious need for mental health services, or medical or psychological assistance, we will, however, guide the young adult to the relevant services. If referral to the secondary service system (i.e., hospital treatment) is urgent, we will refer to the appropriate instance immediately. For milder emerging problems, the researcher will provide the young adult with a list of public and non-governmental organizations and institutions that can be contacted without referral, including telephone counselling, chat forums and open-door services.

## 4 Statistics

### 4.1 Statistical considerations

The analyses of data from previous waves of assessments in this cohort study have shown that the sample is large enough to show group differences of 0.25 Z-scores and larger in tests of neurocognition and social cognition. The size of the sample allows for analyses of mediation via home environment or other environmental exposures from the VIA 7 and the VIA 11 studies and for latent class analyses of trajectories.

Separate statistical analysis plans will be created for each individual study undertaken with the data. However, certain overall principles may be outlined. Differences between the three groups will be analyzed with multivariate and univariate analysis of variance or chi-square test as appropriate. Between-group differences of diagnoses will be evaluated using logistic regression adjusting for the young adult’s sex. Mixed models, Cox regression and latent class growth analysis (LCGA) will be applied in the longitudinal data analyses. Since the data contains sibling pairs, complete independence between observations is not achieved. This will be addressed by either using multilevel mixed models, robust clustered standard errors, or both, as appropriate in each individual study. All analyses will be preregistered. The dimensionality of the data warrants a clearer methodological description. High-dimensional features will be handled using penalized regression (LASSO/elastic net), partial least squares, and random forests, combined within a SuperLearner ensemble framework. Model performance will be evaluated using nested cross-validation to obtain unbiased estimates of out-of-sample prediction accuracy, in line with our established practice in this cohort. We will also clarify *a priori* feature selection and modality-weighting strategies.

Epigenome-wide association analyses: We will perform cross-sectional epigenome-wide association analyses to identify epigenetic markers of brain structure and activation, as well as social cognition, language, olfactory function, measures of hormones and immune function. Apart from additive regression models we will also investigate interaction scenarios between DNA methylation, genetics and environmental exposures with measures of brain structure and functioning in the VIA children as outcomes. Additionally, with the unique access to the longitudinal data on all the above-mentioned traits we will be able to identify DNA methylation markers of brain development trajectories across the 19-year follow-up period (from VIA 0 to VIA 19).

Given the current uncertainty regarding the clinical significance and application of the vast majority of epigenetic data, returning clinically valid, actionable results (e.g., incidental findings) from epigenetic research studies, the establishment of exhaustive criteria for assessing the clinical validity and actionability of epigenetic data is considered premature at this time. We will establish a task force in order to follow the development of validated criteria for returning results from epigenetic research.

## 5 Results

The data collection for the VIA 19 study is expected to be finalized mid-2027, with results published from 2028.

## 6 Discussion

This paper provides a detailed description of the fourth wave of the Danish High Risk and Resilience Study – VIA 19, investigating offspring with familial risk of developing schizophrenia or bipolar disorder. We plan thorough clinical assessments of predefined domains together with MRI, EEG, MEG and PSG. In the previous waves, retention rates were very high, (89.1% and 81.8%in the VIA 11 and VIA 15 study, respectively). We anticipate that our flexible and individual approach will encourage the young adults to participate again.

Given the well documented typical age of illness onset (47), some of the young adults will display symptoms or full clinical presentations of schizophrenia or bipolar disorder at age 19 which is the main focus of this longitudinal cohort study. However, some participants will also have symptoms or diagnoses of other mental illnesses, such as depression, ADHD, autism, anxiety as indicated by our findings in previous assessments (18, 24) and a recent meta-analysis (8). At age 19, we expect the diagnoses to be more evident than at age 15, where symptoms spanning different diagnostic categories may overlap and vary in intensity (124). In the period, from 2012–2024 spanning VIA 7 - VIA 15, there has been a significant increase in referrals to psychiatric services, both before and after age 18, in Denmark (47) as well as an intensified public debate about young people’s poor or compromised mental health, which also took place internationally (124, 125). There is no simple explanation for this increase in mental health problems among young people. However, individuals with a genetic predisposition to mental illness are disproportionately more vulnerable in a society with a more general increase in youth with mental health problems and lack of thrive. The field is complex due to many interacting risk factors. Protective factors are especially crucial but generally less accessible compared with the general population. Mental health problems constitute by far the largest percentage of health problems in adolescence and the first decades of life, with 63-75% of mental illnesses having their onset before age 25 (126). Therefore, more emphasis should be given to how adolescents can obtain resilience (i.e., the ability to cope with and overcome “normal” stress in life and regain good function) and strengthen their mental health, both in general and especially among those with a familial predisposition to reduce suffering and societal loss of resources (124, 127).

The time- and resource-intensive task of following an at-risk cohort over decades is critical for elucidating otherwise invisible behavioral, brain maturational, and clinical changes that can provide new insights into risk and resilience, prediction, target areas for future preventive interventions and personalized treatment strategies. Cohorts with narrow age ranges, such as the VIA cohort, are well-suited for examining inter- and intra-individual developmental changes and differences, as they minimize between-participant age variability and possible cohort effects and thereby may be more sensitive to more subtle developmental changes. We have succeeded in avoiding large dropout rates and achieving very high rates of full completion of the comprehensive test battery. This may be due to the substantial effort we have invested in making every family feel welcome and important, while not offering any intervention. We have sent Christmas and birthday cards to all participants over the years, and we have written a reader-friendly newsletter that has been sent after each wave, informing the participants about the research results in a non-stigmatizing way. We have invited all young adults to share feedback or ask questions, and we have conducted an online focus group interview to hear the participants’ opinions about the project. The overall conclusion from the focus group interview was that the adolescents enjoy being part of the study and that the gift cards, the friendly atmosphere and the assessors’ ability to guide them through the many different tasks encouraged them to participate again (unpublished qualitative data). However, we acknowledge the risk of attrition bias and the challenge in keeping most of the participants engaged in the cohort also in the future.

Knowledge from the cohort has already informed researchers, politicians and clinicians about the importance of including the family perspective and the children in the treatment of the parents. Trajectories can be constructed from data collected at four time points, providing detailed insight into which individuals develop severe illness in early adulthood. We have the opportunity to use advanced longitudinal modeling approaches to better investigate mechanisms underlying vulnerability and resilience for individuals at familial risk of mental illness. Retrospective analysis of the data will enable the identification of risk factors at ages seven, eleven and/or fifteen that could be targeted for intervention. We also aim at developing a prediction model using selected, highly sensitive instruments from the VIA test battery, which could be tested in a population-based study of pre-schoolers at familial risk for mental illness. Such a battery should be brief and lead to direct recommendations for the family. The absence of risk factors would indicate no need for action; the presence of some risk factors would lead to targeted interventions and advice for the family, and the presence of several risk factors would lead to direct referral to integrated interventions.

## Footnotes

1 The CELF-5 results are published with permission from Pearson Sweden AB. Pearson has not been involved in the assessment and is neither responsible for the quality of the results nor for the overall results of the research.
